# Assessment of* Pasteurella multocida* A Lipopolysaccharide, as an Adhesin in an In Vitro Model of Rabbit Respiratory Epithelium

**DOI:** 10.1155/2017/8967618

**Published:** 2017-01-29

**Authors:** Carolina Gallego, Stefany Romero, Paula Esquinas, Pilar Patiño, Nhora Martínez, Carlos Iregui

**Affiliations:** ^1^Laboratory of Veterinary Pathology, Universidad de Ciencias Aplicadas y Ambientales, Calle 222 No. 55-37, Bogotá, Colombia; ^2^Faculty of Science, Pontificia Universidad Javeriana, Carrera 7 No. 43-82, Bogotá, Colombia; ^3^Academic Assistant, Veterinary Medicine Program, Universidad de La Salle, Cra. 7 No. 179-03, Bogotá, Colombia; ^4^Laboratory of Cytogenetics and Genotyping of Domestic Animal UGA, Faculty of Veterinary Medicine, National University of Colombia, Bogotá, Colombia; ^5^Laboratory of Veterinary Pathology, Faculty of Veterinary Medicine, National University of Colombia, Bogotá, Colombia

## Abstract

The role of the* P. multocida* lipopolysaccharide (LPS) as a putative adhesin during the early stages of infection with this bacterium in the respiratory epithelium of rabbits was investigated. By light microscopy and double enzyme labeling of nasal septa tissues, the amount of bacteria attached to the respiratory epithelium and the amount of LPS present in goblet cells at different experimental times were estimated. Transmission electron microscopy (TEM) and LPS labeling with colloidal gold particles were also used to determine the exact location of LPS in the cells. Septa that were challenged with LPS of* P. multocida* and 30 minutes later with* P. multocida* showed more adherent bacteria and more severe lesions than the other treatments. Free LPS was observed in the lumen of the nasal septum, forming bilamellar structures and adhering to the cilia, microvilli, cytoplasmic membrane, and cytoplasm of epithelial ciliated and goblet cells. The above findings suggest that* P. multocida* LPS plays an important role in the process of bacterial adhesion and that it has the ability of being internalized into host cells.

## 1. Introduction

*Pasteurella multocida* is considered the most important causal agent of respiratory diseases in rabbits and in other species; this group of microorganisms is responsible for the largest economic losses in production farm animals [1]. Under production conditions, rabbits are frequently subjected to overcrowding, drastic changes in temperature and humidity, or stress, all of which contribute to the appearance of respiratory pathologies caused by* P. multocida* [2–5].

Bacterial adhesion to the mucosal surfaces of a host results from physicochemical interactions between the pathogens and the host epithelial cells, and the ultimate colonization of deeper layers of the mucosa and the successful establishment of an infection depends on this interaction. Therefore, adhesion is considered the first essential step that a pathogen must overcome to cause disease [6–9]. Such interactions occur between molecules located on the surface of host epithelial cells as well as molecules on the bacteria. Lipopolysaccharide (LPS) is the most abundant superficial constituent of Gram-negative bacteria. This glycolipid molecule is fundamental for the survival of these microorganisms and is responsible for causing severe systemic effects in the hosts, such as endotoxemia; therefore, LPS is considered an important virulence factor of these pathogens [10–13]. However, studies on the involvement of LPS during the first steps of infection, namely, during adhesion and colonization of the surface epithelia before the bacteria causes systemic effects, are rare and most are devoted to other bacterial adhesive structures, such as the fimbria [14–17].

Bélanger et al. [18] proposed that the LPS of* Actinobacillus pleuropneumoniae* would be responsible for the adhesion of the bacterium to the respiratory epithelial cells of swine; later, studies of* E. coli, Salmonella enterica* subsp.* enterica* serovar Typhimurium,* Helicobacter pylori, Klebsiella pneumonia,* and* Pseudomonas aeruginosa* showed that it was possible to block the adhesion of the corresponding microorganisms to different cell surfaces by suppressing or modifying the structure of their respective LPS molecules and by employing monoclonal antibodies against the O antigen of the molecule [16, 17, 19–23].

Recently, Bravo et al. [16] showed that the core of* S*.* typhimurium* LPS plays an important role in the interaction of the bacteria with HeLa epithelial cells, as well as BHK and IB3 cells lines. The inner core of the molecule has Glc I and Gal I residues, which are essential for adhesion and entry of the microorganism into epithelial cells in vitro. The authors speculate that a lectin type receptor could be involved in the interaction of the host cells with the outer core structure, which is composed of (Gal I *α* 1-3 Glu) [16].

In addition, a role for* Helicobacter pylori* LPS O antigen in adhesion and colonization of the gastric mucosa of mice has also been proposed. Moreover, it has been shown that the core of* H. pylori* LPS is involved in pathogen binding to laminin, an extracellular matrix and basement membrane glycoprotein of the epithelium that is essential for the process of bacterial adhesion [17].

On the other hand, few reports document the mechanism by which LPS is internalized into the cytoplasm of the host cells; caspases 4, 5, and 11 are considered intracytoplasmic receptors for* E. coli* and* S. typhimurium* LPS in macrophage cell cultures and somatic cells such as mouse enterocytes in which they induce pyroptosis and apoptosis [23]. In addition, LPS can be internalized by the lipopolysaccharide binding protein (LBP) into the cytoplasm of monocytes and macrophages, as well as by nonimmune human cells such as the HEK293 cell line. These findings have led to the hypothesis that LPS plays an important role in the regulation of intracellular pro- and anti-inflammatory signals [24].

In* P. multocida*, different studies reported the significance of several surface structures such as adhesins, including highly hydrated polyanionic polysaccharides on the capsule that are covalently bound to the surface of bacteria through phospholipids or lipid A. Other structures also include outer membrane proteins (OMPs), which may serve as adhesins or invasins or participate in the formation of biofilms such as type IV fimbriae, the fibronectin binding protein, and filamentous hemagglutinin [25]; however, no study has been devoted to exploring the role of* P. multocida* LPS as an adhesion molecule. For the first time, we show that the LPS of* P. multocida* significantly increases the adhesion of the microorganism to the apical surface of the respiratory epithelium of the rabbit nasal septum and that the increased number of adhered microorganisms causes more severe damage to the host cells. We also show that LPS is internalized into the cytoplasm of respiratory cells and that goblet cells play an important role during these first steps of infection.

## 2. Materials and Methods

### 2.1. *Pasteurella multocida* Strain

*Pasteurella multocida* A strain 001 was obtained from turbinates, trachea, and lungs of rabbits with signs of rhinitis and pneumonia from farms in the Sabana de Bogotá (Colombia). The organism was grown in brain heart infusion (BHI) agar; gray nonhemolytic colonies with a round morphology were selected. The colonies were identified as bipolar-staining, Gram-negative coccobacilli and were shown to be catalase-, oxidase-, indole-, ornithine decarboxylase-, glucose-, sucrose-, and mannitol-positive using biochemical tests. The colonies did not grow on MacConkey agar and were urease-negative [1]. Molecular identification of* P. multocida* of capsular serogroup A was performed by expanding the sequence of the hyaD cap gene locus with the following primers: F: 5′ TGC CAA AAT CGC AGT CAG 3′; R: 5′ TTG CCA TCA TTG TCA GTG 3′ [26].

### 2.2. Extraction, Purification, Quantification, and Biological Activity of* P. multocida* LPS

LPS was extracted using the phenol/hot water method [30].* Pasteurella multocida* (5.8 g) were resuspended in 29 mL of ultrapure water. The aqueous phase was dialyzed against sterile water and then lyophilized. The sample was reconstituted in 10 mL of 0.1 M Tris buffer containing 0.15 M NaCl and 1 N HCl, pH 7, and treated with RNase (0.5 mg/mL), DNase (0.05 mg/mL), and 100 *μ*L of 4 mM MgCl_2_ for 2 hours at 60°C. The sample was then treated with proteinase K (0.05 mg/mL) and 100 *μ*L of 1 mM CaCl_2_ for 18 hours at 37°C. The extract was frozen at −80°C and lyophilized for 48 h. A mass of 70.4 mg was obtained and was then subjected to gel permeation chromatography; briefly, the extract was resuspended in 5 mL of 0.05 M Tris buffer containing 1.5% sodium deoxycholate and 1 mM EDTA, pH 9.5, and passed through a GE Healthcare HiPrep™ 16/60 Sephacryl™ S-200 HR column (Sigma Aldrich, St. Louis, MO, USA). Fractions of 2.5 mL were collected at a flow rate of 0.8 mL/min and measured at 220 nm, 260 nm, and 280 nm using spectrophotometry. At each stage of purification and characterization, aliquots were sampled and were analyzed with a spectrophotometer by scanning between 200 nm and 600 nm (NanoDrop 2000 Spectrophotometer Thermo Scientific, Wilmington, DE, USA).

The resolving gel for electrophoresis was prepared at 12%, the stacking gel was prepared at 4%, and the electrophoresis conditions were 0.03 A/45 min and 0.02 A/1 h, respectively. The lyophilized fractions were reconstituted in pyrogen-free water to a final concentration of 1 mg/mL, prepared in 1x Laemmli buffer, and heated at 95°C for 5 minutes before they were added to the gel. Staining was performed with silver nitrate and Coomassie blue [31].

The amount of polysaccharide in the extract was quantified using the Purpald test; a reference curve was constructed with a standard of 3-deoxy-*α*-D-mannooctulosonic acid (Kdo) (Sigma Co., St. Louis, MO, USA) in concentrations ranging from 0.8 mM to 0.025 mM. Commercial* Escherichia coli 0111:B4, S. typhimurium* and* Rhodobacter sphaeroides* DSM 158 LPSs were diluted to two concentrations: 0.4 mg/mL and 0.2 mg/mL. Different concentrations of the* P. multocida* LPS fractions were used, ranging from 1 mg/mL to 0.125 mg/mL. Duplicates of each sample were placed in a 96-well microplate containing 32 mM sodium periodate; the plates were incubated at room temperature for 25 min. Subsequently, 136 mM Purpald reagent was added to each well and incubated for 20 min at room temperature. Immediately, 64 mM of sodium periodate was added and incubated at room temperature for 20 min. Then, 20 *μ*L of 2-isopropanol was added. Spectrophotometry was performed at 550 nm.

The sterility of the LPS was confirmed with cultures in agar BHI in the presence and absence of 5% ovine blood, which was incubated at 37°C and observed daily for six days.

The biological activity of LPS was determined in mice by evaluating its pathogenic effects. Twenty-five *μ*g of LPS diluted in 100 *μ*L of PSS was intraperitoneally inoculated in five mice; 125 *μ*L of PSS was also IP injected in 5 additional mice as a negative control. After eight hours, the animals were euthanized and the lungs and liver were evaluated by histopathology.

### 2.3. Simultaneous and Sequential Exposure of Rabbit Nasal Septa Explants to* P. multocida* and to LPS (H&E)

The nasal septa of rabbit fetuses were cultured ex vivo using the methods reported by [32, 33]. Briefly, fetuses were obtained by cesarean on gestational day 26 under anesthesia and were immediately euthanatized by medullar sectioning; two sequential approximately 2 mm thick cross sections of the nasal cavity were maintained in Dulbecco's minimal essential medium (MEM) during the experiment. All procedures were approved and authorized by the Bioethics Committee of the Faculty of Veterinary Medicine and Animal Science, National University of Colombia (Act 006/2010).

*Experimental Design*. The tissues were treated as described in Table 1.

The tissues were immersed in 10 mL of MEM in 5 cm wide × 2 cm high Petri dishes; the tissues were incubated in a humid chamber at 37°C with a 5% CO_2_ atmosphere for 2 h. At the appropriate times, the tissues were immersed in McDowell and Trump fixative (commercial 4% formaldehyde and 1% glutaraldehyde) diluted in Sorenson's sodium phosphate buffer.

The lesions on the respiratory epithelium were evaluated with a 100x objective and were semiquantitatively assessed. The severity and extent of the lesions were graded using the scale reported by Bernet et al. [29] (Table 2).

The following changes or lesions were evaluated: desquamated cells (DC), activity of goblet cells (AGC), cilia loss (CL), the presence of intracytoplasmic vacuoles (IV), and dead cells (DC).

### 2.4. Evaluating the Amount of* P. multocida* LPS on the Apical Surface of Respiratory Epithelial Cells and within the Cytoplasm of Goblet Cells, as well as the Number of* P. multocida* That Adhered to the Respiratory Epithelium (LH-IIP)

*P. multocida* and* P. multocida* LPS were simultaneously detected on the respiratory epithelium of nasal septa by a double labeling technique using indirect immunoperoxidase (IIP) for the bacterium and lectin histochemistry (LH) for LPS. Briefly, polyclonal ovine antibodies labeled with horseradish peroxidase (HRP) were employed to detect* P. multocida*; a specific commercial* Limulus polyphemus* lectin (LPA) conjugated with alkaline phosphatase was added to the nasal explants (alkaline phosphatase-conjugated* Limulus polyphemus* lectin, horseshoe crab, EY Laboratories Inc., San Mateo, CA, USA). This lectin specifically binds the 2-keto-3-deoxyoctanate (KDO) sugar of the core of LPS [34]. The peroxidase technique was performed first, and then the tissues were incubated with the LPA lectin.

### 2.5. Analysis of the Images of LPS Lectin Histochemistry and Indirect Immunoperoxidase Staining of* Pasteurella multocida*

The program ImageJ version 1.41 GPL (General Public License), which is available for all systems and platforms and is widely used for analyzing biomedical images, was implemented to evaluate the quantity of LPS on the apical surface of epithelial cells and the number of* P. multocida* that had adhered to the ciliated border of the respiratory epithelium and within the cytoplasm of goblet cells [35].

### 2.6. Transmission Electron Microscopy

The tissues were dehydrated in an ascending ethanol series from 50% to 90% and were then embedded in LR White resin. Ultrathin sections (90 to 100 nm) were cut and mounted on nickel grids or nickel-covered 200 mesh Formvar grids; the samples were rehydrated in distilled water. Nonspecific sites were blocked with 10% bovine serum albumin in PBS for 15 minutes, incubated on a drop of lectin* Limulus polyphemus* diluted 1 : 3 in a buffer solution, and conjugated to 5 nm wide colloidal gold particles for 30 minutes at 37°C. The grids were washed and contrasted with 1% uranyl acetate for 3–5 minutes and observed with a Jeol 1400 Plus transmission electron microscope at 80 kV [36]. No IIP was necessary because bacteria are more easily visible with this technique than with light microscopy.

### 2.7. Statistical Analysis

For the model assumptions, the Shapiro-Wilk test was used to determine the normality of the errors and Levene's test was used to determine the homogeneity of the variance. Analysis of variance (ANOVA) with a confidence interval of 95% and Tukey's multiple comparison test were used to determine the differences between treatments [37].

## 3. Results

### 3.1. *Pasteurella multocida* Strain

According to the taxonomic tools of SeqMatch classification from the RDP, the comparison of the BlastN of Greengens, and the basic alignment in NCBI, the strain of* P. multocida* used in this study shares 99% identity with the genus and species* Pasteurella multocida* subsp.* multocida*.

### 3.2. Extraction, Purification, Quantification, and Biological Activity of* P. multocida* LPS

**Figure 1:**
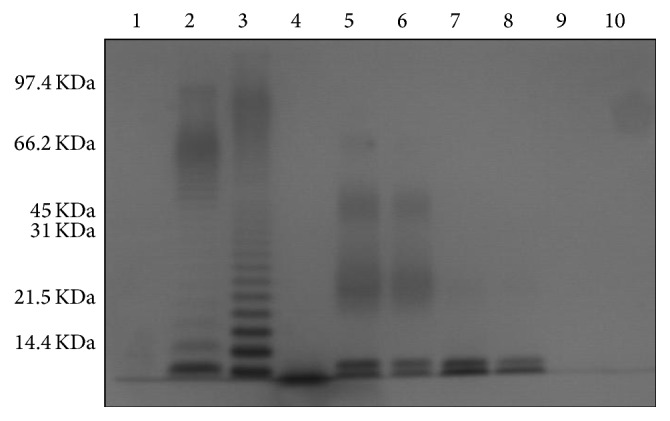
Electrophoretic profile of LPS of* P. multocida* A UN001: 1, low molecular weight marker; 2, commercial LPS of* Escherichia coli* 0111:B4 (InvivoGen, San Diego, CA, USA) (0.06 mg/well); 3, commercial lipopolysaccharide of *S. typhimurium* ATCC 7823 (Sigma Co., St. Louis, MO, USA) (0.1 mg/well); 4, commercial LPS of* Rhodobacter sphaeroides* DSM 158 (InvivoGen, San Diego, CA, USA); 5, LPS of* P. multocida* A UN001 (0.06 mg/well); 6, LPS of* P. multocida* A UN001 (0.03 mg/well); 7, LPS of* P. multocida* A UN001 (0.06 mg/well); 8, LPS of* P. multocida* A UN001 (0.06 mg/well); 9, LPS of* P. multocida* A UN001 (0.06 mg/well); 10, LPS of* P. multocida* A UN001 (0.03 mg/well).

A purified smooth chemotype of* P. multocida* LPS with the presence of KDO and heptoses in its core was obtained. It was also protein-free (Figure 1).

### 3.3. Simultaneous and Sequential Exposure of Rabbit Nasal Septa Explants to* P. multocida* LPS (H&E)

Nasal septa that were only challenged with* P. multocida* showed moderate cell desquamation and increased goblet cells activity, consisting of dilatation of the cytoplasm. Most of these cells exhibited this change. Slight cilia loss was present in ciliated cells; in some, the cytoplasm was vacuolated, and others were dead.

The changes and lesions in the respiratory epithelium of treatments 3 and 4, that is, simultaneous exposure to* P. multocida* LPS and* P. multocida* and exposure to* P. multocida* LPS followed by* P. multocida* 30 min later, were more severe. These changes consisted of desquamated cells and the presence of detritus in the septal lumen. In addition, increased dilatation of the cytoplasm of goblet cells and a notorious extrusion of their secretion were common findings in many others; moderate loss of cilia, cytoplasmic vacuolization, and death of the ciliated cells were more abundant. No such changes or lesions were observed in the nasal septa of the control group (treatment 1) that was only incubated with MEM without bacteria or LPS (Table 3).

### 3.4. Evaluating the Amount of* P. multocida* LPS on the Apical Surface of Respiratory Epithelial Cells and within the Cytoplasm of Goblet Cells, as well as the Number of* P. multocida* That Adhered to the Respiratory Epithelium (LH-IIP)

The quantity of* P. multocida* LPS on the apical surface of goblet cells and ciliated cells and within their cytoplasm and the number of bacteria that adhered to the respiratory epithelium are recorded in Table 4. The results correspond to the mean of pixels of seven repetitions for each treatment; the colorimetric information is transformed to numeric information under the color model RGB (Red-Green-Blue). Based on the previous analysis, the intensity and extent of the labeling for LPS and for* P. multocida* were increased in the treatment group in which* P. multocida* LPS and* P. multocida* were administered simultaneously, followed by the challenge with* P. multocida* LPS and 30 min later with* P. multocida*, the challenge with* P. multocida* and 30 min later with its LPS, and finally the tissues that were only exposed to* P. multocida*.

**Figure 2:**
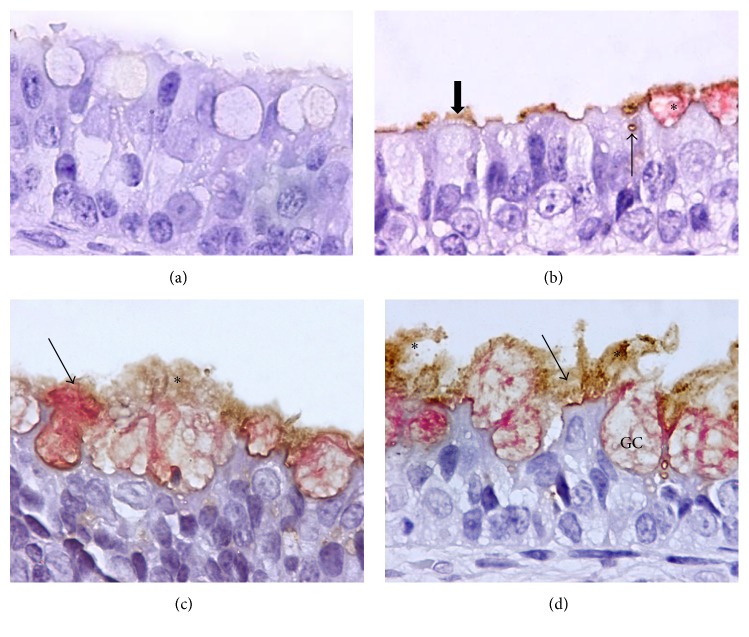
Simultaneous and sequential exposure to* P. multocida* and to its LPS and to* P. multocida* alone in nasal septa explants: LH and IIP. (a) Tissue cultures that were not exposed to LPS or* P. multocida*; ciliated cells and goblet cells were not labeled. (b) Tissues that were only exposed to* P. multocida* (thick arrow). LPS is visible within the cytoplasm of goblet cells (*∗*); faint LPS labeling lines the apical border of apparently ciliated cells that have lost their cilia; an apparent brown color is located intracytoplasmically in a nonidentifiable cell (thin arrow). (c) Simultaneous exposure to* P. multocida* LPS and* P. multocida* (*∗*). Most of the LPS labeling is located within the cytoplasm of goblet cells; several goblet cells are extruding their content along with LPS (arrow). (d) Tissues that were exposed to* P. multocida* LPS followed by* P. multocida* (*∗*) 30 min later show a similar appearance and signs as in (c); note the clearer interface between LPS and the bacteria (thin arrows); GC: goblet cell. H&E: 100x.

Positive* P. multocida* staining was brown and had a granular appearance, whereas the LPS staining was reddish and of diffuse character; in experiments 2 and 3 (Table 1), both colors were adjacent at the interface (Figure 2). On the other hand, in tissues that were only exposed to* P. multocida* (Figure 2(b)), the brown color was mainly observed on the ciliated border of the corresponding cells and free in the lumen of the tissue. Interestingly, the characteristic reddish LPS staining was also visible within the goblet cells and apparently lining the apical border of nonidentifiable epithelial cells. Moreover, both colors, the brown* P. multocida* and the reddish LPS, were also observed in close proximity to each other in the treatment groups where both the bacterium and its LPS were administered, albeit to a lesser extent (Figures 2(c) and 2(d)).

No positive labeling was observed for either antigen,* P. multocida* or its LPS, in tissue that was not exposed to the antigens (Figure 2(a)).

A confidence interval of 95% (*α* = 0.05) was used for all statistical analyses.

### 3.5. Transmission Electron Microscopy (TEM)

Tissues from the negative control (treatment 1) that were only cultured with MEM, explants that were only exposed to* P. multocida* (treatment 2), explants that were exposed to LPS and then to* P. multocida* 30 minutes later (treatment 4), and explants that were only treated with LPS (treatment 6) were processed for TEM and LH (Table 1).

A* Pasteurella multocida gatF* mutant (AL2116) was also used that was unable to assemble the external core of LPS beyond Glc IV; GatF is the galactosyltransferase which adds Gal I to the 4th position of the Glc IV [38]; in these experiments no bacteria were seen to adhere to the respiratory nasal epithelium; moreover, LPS staining with* Limulus polyphemus* colloidal gold-labeled lectin was very poor or was completely absent, and no ultrastructural changes in the respiratory epithelium were observed after challenge with the mutant strain (kindly donated by Dr. Ben Adler, Bacterial Pathogenesis Research Group, Department of Microbiology, Monash University).

**Figure 3:**
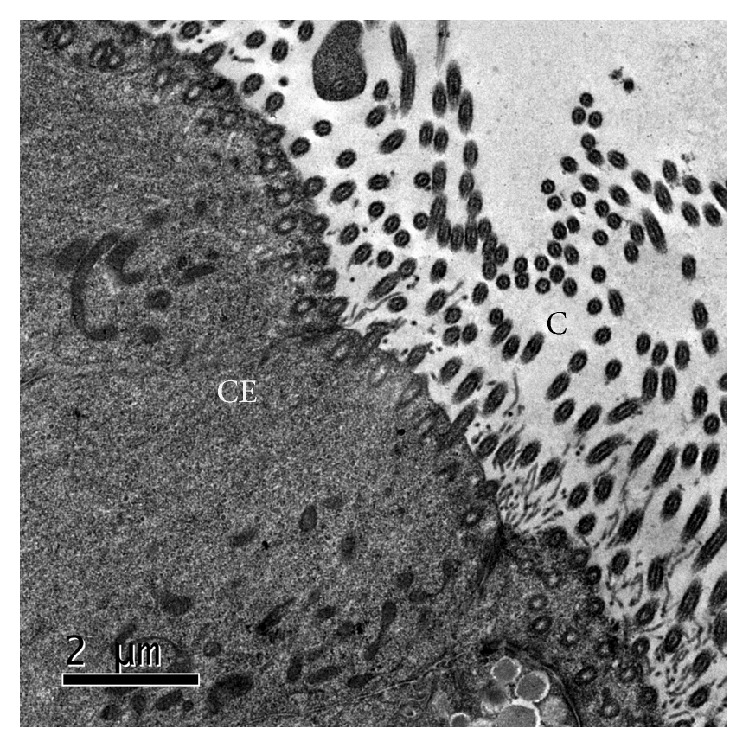
Respiratory epithelium of the nasal septum (treatment 1). Negative control (treatment 1). Ciliated epithelial cell (CE) and cilia (C). MET and gold-labeling of LPS using a lectin of* Limulus polyphemus*.

No ultrastructural changes or positive labeling with colloidal gold was observed in tissues that were not treated with* P. multocida* or its LPS (Figure 3).

### 3.6. Location of the Ultrastructural Changes in the Nasal Septa Caused by* P. multocida* at Two Hours after Incubation (Treatment 2)

**Figure 4:**
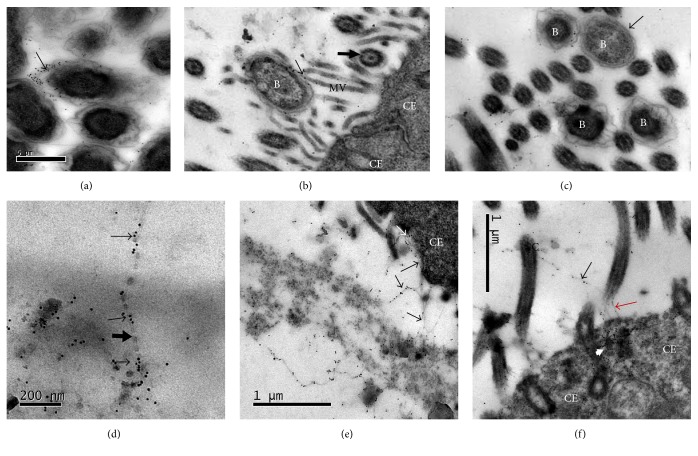
Respiratory epithelium of the nasal septum that was exposed to* P. multocida* A UN001 (treatment 2). MET and gold-labeling of LPS using a lectin of* Limulus polyphemus*. (a) Bacterial structure with positive labeling on the outer membrane (thin arrow). Note the abundant capsule that surrounds each bacteria. Bacterial culture. (b) Note the interface of the LPS between the bacterium (B) and microvilli (thin arrow). Some LPS is free in the lumen admixed with amorphous material but is also visible at the surface of some microvilli. Microvilli (MV) of a ciliated epithelial cell (CE) and cilia (thick arrows). (c) This particular bacterium has lost its capsule (arrow) compared with three other bacteria (B). (d) Thread-like bilaminar structure (thick arrow) located in the lumen of the nasal septum that is positively labeled with LPS (thin arrows). (e) Multiple thread-like LPS-positive bilaminar structures are associated with the cytoplasmic membrane and microvilli of an ciliated epithelial cell (CE) (black arrows); some gold particles seem to ingress into the cell (white arrow). (f) A bilaminar structure bridges two cilia (black arrow), and similar thread-like bilaminar structures bridge the cilia and the cytoplasm of the same cell where the base of a cilia was not included (red arrow); large numbers of gold particles are already present within the cytoplasm of the same cell (arrowheads).

In nasal septa that were only exposed to* P. multocida* for two hours, the bacteria adhered to the microvilli and cilia of the corresponding cells. In bacterial culture, the external bacterial membrane and, even more so, the capsule were LPS-positive as they reacted with colloidal gold-labeled lectin (Figure 4(a)). In tissue culture, LPS is localized at the interface of the bacterium and the cell membrane of microvilli of ciliated cells, which are also LPS-positive (Figures 4(b) and 4(c)). Moreover, in the lumen of the organ, thread-like structures resembling a cellular bilayer membrane were also evident and were associated with the microvilli and cilia and even within the cytoplasm of epithelial cells (Figures 4(d), 4(e), and 4(f)); many of these threads spanned the distance between cilia, microvilli, and the apical membrane, building an apparent scaffold. No or only a few ultrastructural changes were observed in the epithelial cells of these septa.

### 3.7. Location of the Ultrastructural Changes in Epithelial Cells Caused by Exposure to* P. multocida* LPS and Then to* P. multocida* 30 Minutes Later (Treatment 3)

**Figure 5:**
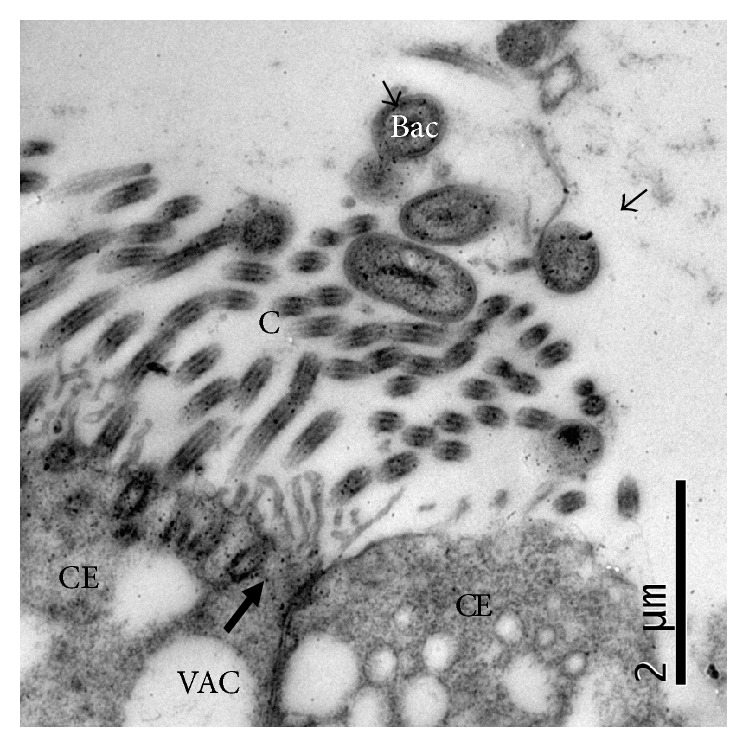
Respiratory epithelium of the nasal septum that was exposed to* P. multocida* LPS and then to* P. multocida* 30 min later (Treatment 4). MET and gold-labeling of LPS using a lectin of* Limulus polyphemus*. Bacterial structures (Bac) were positively labeled on their outer membrane (arrows) and were associated with the cilia (C) of a ciliated epithelial cell (CE); all microorganisms have lost their capsule. Large numbers of colloidal gold particles are visible at the apical cytoplasm of the cell. Cytoplasmic vacuolization (VAC).

Nasal septa that were sequentially exposed to* P. multocida* LPS and to* P. multocida* 30 min later showed increased numbers of bacteria attached to the cilia of epithelial cells; positive labeling for LPS (colloidal gold nanoparticles of 5 nm in diameter) was also visible on the outer membrane of the bacteria, as well as in the cilia and cytoplasm of ciliated cells. The bacteria lost their capsule. Moderate ultrastructural changes such as epithelial cell cytoplasmic vacuolization were more evident in this experiment (Figure 5).

### 3.8. Location of the Ultrastructural Changes in Nasal Septa Induced by* P. multocida* LPS at Two Hours after Incubation (Treatment 6)

**Figure 6:**
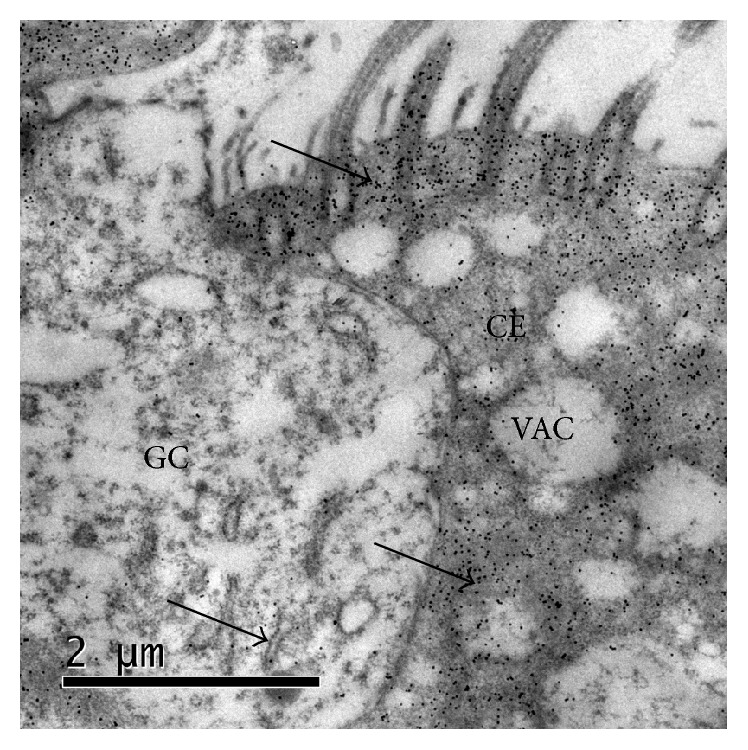
Respiratory epithelium of the nasal septum that was exposed to* P. multocida* AUN001 LPS (treatment 6). MET and gold-labeling of LPS using a lectin of* Limulus polyphemus*. Intense* P. multocida* LPS-positive labeling is associated with the cilia, cytoplasmic membrane, and cytoplasm (arrows) of a ciliated epithelial cell (CE) and with a goblet cell; severe intracytoplasmic vacuolization (VAC) is observed.

Intense labeling in the cilia and within the cytoplasm of goblet cells and ciliated cells was observed in nasal septa that were exposed to* P. multocida* LPS for two hours. Severe cytoplasmic vacuolization was observed in epithelial cells that were only exposed to LPS (Figure 6).

## 4. Discussion

The LPS of Gram-negative bacteria is considered one of the main virulence factors of these pathogens, causing severe systemic damage in the interaction with their hosts [39–41]; a similar role has been recognized for the LPS of* P. multocida* [39–42]. Accordingly, most studies with these molecules have been devoted to understanding the systemic effects caused by LPS once it reaches the circulatory stream [11, 41, 43, 44]. In contrast, much less effort has been dedicated to investigating the role of LPS in its respective bacteria during the first phases of an infection and attachment to the mucosal surfaces of the corresponding hosts [45]. In an in vitro model, we provide morphological evidence that* P. multocida* A LPS mediates the adherence of the bacterium to the apical surface of the nasal respiratory epithelium in rabbits; to our knowledge, this finding has not been reported for this pathogen.

It is considered that a Gram-negative bacterium possesses approximately 3.5 × 10^6^ LPS molecules that occupy an area of 4.9 *μ*m^2^ on its outer membrane. Given that the approximate surface of one of these microorganisms ranges from 6 to 9 *μ*m^2^, the LPS would cover approximately 75% of its surface, constituting the main component of the outer membrane of these agents [11, 46, 47]. Taking into account the amount of LPS on the surface of a Gram-negative bacterium, its privileged localization on the surface of the microorganism, and its rich content of carbohydrates, some of which have adhesive properties, and so forth, it is surprising that the role of this molecule during the first steps of infection with these microorganisms has not been more thoroughly investigated. Recently, the role of the core oligosaccharide of* Helicobacter pylori* LPS in mediating the adhesion of the bacterium to laminin, an extracellular matrix glycoprotein in host basement membrane that is believed to be essential in the cellular adhesion process, was suggested [17]. In the same vein, Bravo et al. [16] showed that the outer core of* S. typhi* LPS, which is composed of Glc I, Gal I, and Glc II, was required for effective adhesion and bacterial entry into HEp-2 cells.

Based on these findings and on our own previous results [48] we proposed that the LPS of* P. multocida* could be a good adhesive candidate for mediating pathogen attachment to the respiratory epithelium of rabbits during the first phases of infection. Accordingly, by simultaneously and sequentially exposing the nasal septa of fetal rabbits to* P. multocida* LPS and to the bacterium, it was possible to show that the LPS of* P. multocida* significantly increases the amount of* P. multocida* that attaches to the respiratory epithelial cells of the nasal septa compared with the septa that were only exposed to the pathogen. This result was confirmed by TEM experiments, which showed that higher numbers of bacteria adhered to the cilia of epithelial cells when the septa were previously exposed to LPS (Figure 5).

Using lectin histochemistry (LH),* P. multocida* LPS was more visible within the cytoplasm of goblet and ciliated cells, on their apical border, surrounding the cilia and in the extracellular medium. This result was confirmed by TEM. On the other hand, as shown in Figure 2, larger numbers of bacteria adhered to goblet cells (*P* < 0.05); moreover, in the two treatments where nasal septa cultures were simultaneously and sequentially exposed to the LPS and the bacterium, the severity of the lesions in the respiratory epithelial cells was significantly increased (*P* < 0.001), compared with the septa that were only treated with* P. multocida* or were treated with the microorganism and then its LPS 30 min later (Table 3).

However, and perhaps more importantly, an unexpected finding in this study was that the explants that were only exposed to* P. multocida* were also positively labeled for its LPS by LH, understandably, in a lower amount compared with the explants that were exposed to the molecule and the bacterium. This staining included the same structures as when the LPS and the bacterium were added simultaneously or sequentially, that is, within the cytoplasm of goblet cells and ciliated cells, as well as a thin film over their respective apical membrane. Previous findings were corroborated by TEM, where, in addition to the surface of the bacterium, the molecule seems to join the microorganism to the microvilli and cilia of ciliated cells (Figures 4 and 5). More detailed location and relationship of the molecule with the apical membrane of goblet cells and ciliated cells were shown by this technique, where it was observed to adhere to the cilia, the microvilli, the apical membrane, or even spanning two neighboring cilia (Figure 4), building a mesh-like structure that may favor bacterial adherence; finally, the molecule reached the cytoplasm of the epithelial cells. One possible explanation for this finding would be that* P. multocida* spontaneously releases its LPS from its outer membrane from the very first incubation time [49, 50] or from the very first moment of its interaction with the respiratory epithelium, as shown in this work. Interestingly, the experiment in which the most significant number of bacteria adhered was the experiment in which nasal septa cultures were simultaneously exposed to the LPS and to* P. multocida*. This finding suggests that, under natural conditions, the adherence of the bacterium to the respiratory epithelium would occur in a matter of minutes. This result fits well with the experimental results of infection in rabbits, which develop clinical signs after two hours. In any case, the unexpected release of LPS under the experimental conditions indirectly favors the suggested adhesive role of the* P. multocida* LPS under natural conditions.

Notably, in this work, the microorganisms display an abundant capsule immediately before they are added to the nasal septa cultures compared to the bacteria adhering to microvilli or cilia, which have completely lost their capsule, or when only a small amount of the capsule remains (Figures 4 and 5). The presence of a capsule on Gram-negative and Gram-positive bacteria has repeatedly been reported as an impediment for their ability to adhere to mucosal surfaces. One mechanism proposed for this interference is that the capsule masks adhesin(s) that might be located deeper on the bacterial surface [10, 51, 52]; this mechanism works well in the case of* P. multocida* in this study.

Lipopolysaccharide-positive labeling of the same* P. multocida* A within the cytoplasm of goblet cells in the same experimental model used in this research has been already reported [32]; the only difference was that the previous study labeled the LPS with polyclonal antibodies instead of with* Limulus polyphemus* lectin, as in this work. Using TEM, Esquinas et al. [53] showed that myelin-like structures were contained within the vacuoles in the cytoplasm of ciliated cells and suggested that these structures might correspond to* P. multocida* LPS; this study confirms that the LPS of* P. multocida* effectively reaches the cytoplasm of these cells and that cytoplasmic vacuoles are formed in these cells, which may be caused by this molecule. There are some possibilities to explain how the molecule reaches the cytoplasm of goblet cells and ciliated cells. One is that the LPS of P*. multocida,* similar to the LPS of other Gram-negative pathogens, would be recognized by free, mucus- and glycocalyx-located CD14 receptors that would be internalized by activation of the TLR4/MD2 complex [24, 54–56]. Another soluble receptor is LPS binding protein (LBP); this receptor binds LPS in the blood plasma and transfers it to cellular receptors, thus increasing the activation of cells that lack mCD14 [57, 58]. The mechanisms for the internalization and intracellular transport of the endotoxin are not clear, but some authors propose that it travels after interacting with CD14 [59], whereas others suggest that the internalization involves a clathrin-dependent pathway through which it is transported via lipid rafts to the Golgi apparatus to bind intracellular TLR4 [15, 60–62].

Recent studies report that LBP catalyzes the intercalation of LPS into the reconstituted phospholipid bilayers, providing an additional mechanism of LBP-mediated transport of LPS into host cell membranes and the cytoplasm of human monocytes and macrophages [24]. In this regard, it is quite interesting that the LPS of* P. multocida* adopted a thread-like bilaminar appearance and was observed in direct interaction with the cell membrane of the ciliated cells and their cilia in this work (Figures 4(d), 4(e), and 4(f)), which could be evidence of the intercalation described by [24]. It should be noted that these bilamellar structures were only observed in the LPS that was spontaneously released by the bacteria (treatment 2, Table 1) and were not observed in experiments that used purified LPS (treatments 3 and 4, Table 1), which is more similar to the natural conditions of the infection.

In experiments with purified LPS (treatments 3 and 4, Table 1), it was found that LPS induced ultrastructural changes of the respiratory epithelium, such as cytoplasmic vacuolization and dilated interepithelial spaces of the nasal septa; the changes induced by* P. multocida* LPS alone were more severe than the changes caused by the bacterium alone (Figures 5 and 6). This finding could be explained by the LPS dose to which the explants were exposed; surely, the amount of LPS that is spontaneously released by the bacteria is much lower than the amount of purified LPS used in the experiments. Studies exist that show that the effect of LPS is dose-dependent. It is possible that, in the first instance, the pathogen does not try to activate mechanisms that lead to tissue damage but induces intracellular signals that stimulate the immune system. Some authors argue that this upregulation may occur in the body in response to high doses of LPS or by a state of hyperreactivity to LPS. On the other hand, when there is contact with low levels of LPS, beneficial effects on the host such as the development of resistance to infections may be triggered [24, 63–66].

Yan et al. [67] proposed a mechanism by which LPS upregulates mucin genes such as MUC5AC and MUC2 expression in human epithelial cells; they demonstrated that bacterial LPS utilizes reactive oxygen species (ROS) for transmitting signals to provoke the host defense response and upregulate MUC5AC mucin expression. Gram-negative bacteria would use some substrates present in glycoproteins produced by goblet cells as the first binding site to initiate adhesion. It is proposed that the mechanism would start from metaplasia of mucus-producing cells induced by proinflammatory cytokines such as TNF alpha released from leukocytes by the action of LPS [32, 67–71].

More recently, the PLUNC (palate, lung, and nasal epithelium clone) protein, which is related to the LPS binding protein (LBP) family, has been demonstrated in epithelial cells, goblet cells, and glandular airway cells [72, 73], and it has been suggested that it is stored and released from the granules of neutrophils [74]. PLUNC recognizes and binds to LPS, and it is proposed that PLUNC may play a role in the innate immune response of the upper airways [73, 75]. Consistent with these findings, our results might suggest that the positive labeling for* P. multocida* LPS mixed with mucus, in the cytoplasm of inflammatory cells that accompany this mucus, and inside the glandular and epithelial cells could be explained in part by the binding of the endotoxin to this substance [45].

It can be concluded that the LPS of* P. multocida* A UN001 plays an important role in the adhesion of bacteria to the rabbit respiratory epithelium by increasing the number of adherent bacteria in the presence of LPS. Lipopolysaccharide is spontaneously released by the bacteria and interacts with structures such as the cilia, microvilli, and cytoplasmic membrane to achieve internalization into the cytoplasm of epithelial ciliated and goblet cells. These results should be the foundation of new research focused on determining exactly which components of* P. multocida* LPS interact with the host cell not only to advance the understanding of the pathogenesis of this disease but also to propose mechanisms to inhibit this interaction.

## Figures and Tables

**Table 1:** Experimental protocol for exposing fetal rabbit nasal septa to *P. multocida* and LPS: IIP (indirect immunoperoxidase), LH (lectin histochemistry), TEM (transmission electron microscopy), and C (−) (negative control)^*∗*^ [27, 28].

Treatment	Negative control		*P. multocida*		LPS + *P. multocida* simultaneously	LPS + *P. multocida *30 min later		*P. multocida *+ LPS 30 min later	LPS
	Treatment 1		Treatment 2		Treatment 3	Treatment 4		Treatment 5	Treatment 6
Technique	IIP-LH	TEM	IIP-LH	TEM	IIP-LH	IIP-LH	TEM	IIP-LH	TEM
Number of explants	7	3	7	3	7	7	3	7	3
Dose^*∗*^	MEM	MEM	1 × 10^8^ cfu/mL	1 × 10^8^ cfu/mL	10 *µ*g/mL + 1 × 10^8^ cfu/mL	10 *µ*g/mL + 1 × 10^8^ cfu/mL	10 *µ*g/mL + 1 × 10^8^ cfu/mL	1 × 10^8^ cfu/mL + 10 *µ*g/mL	10 *µ*g/mL
Exposure time	2 h	2 h	2 h	2 h	2 h	2 h	2 h	2 h	2 h

**(a):** 

Grade	Severity	Description
−	Absent	(i) Cells with normal activity (ii) Normal architecture is readily visible
+	Light	(i) Cells with scant activity (ii) Lesions are observed with some difficulty
++	Moderate	(i) Cells with moderate activity (ii) Lesions are easily observed, with normal architecture still visible
+++	Severe	(i) Cells are very active (ii) Lesions are easily observed; the normal architecture is not identified

**(b):** 

Extension	Description
Focal	F: lesion in an area comprising less than 10% of the section
Multifocal	M: lesions in more than two areas, each comprising less than 10% of the section
Diffuse	G: multiple areas or an area greater than 50% of the section

**Table 3:** Grading of the lesions after each treatment (H&E): desquamated cells (DQ); increased goblet cells activity (AGC); cilia loss (CL); presence of intracytoplasmic vacuoles (IV); and dead cells (DC).

Treatment	DQ	AGC	CL	IV	DC
(1) Negative control	−	−	−	−	−
(2) Exposure to *P. multocida*	++	++	+	+	+
(3) Simultaneous exposure to *P. multocida* LPS and *P. multocida*	+++	+++	++	++	++
(4) Exposure to *P. multocida* LPS followed by *P. multocida *30 min later	+++	+++	++	++	++
(5) Exposure to *P. multocida *followed by *P. multocida *LPS 30 min later	++	++	++	++	++
(6) Exposure to LPS of *P. multocida*	+++	+++	+	+	+

**Table 4:** Mean estimated quantity of LPS on the apical surface of epithelial cells and within the goblet cells and the number of bacteria that adhered to the ciliated border (mean of 7 repetitions for each experiment).

Treatment	Estimated number of bacteria (pixels) (mean ± SD)	Estimated LPS quantity (pixels) (mean ± SD)
(1) Negative control	0	0
(2) *P. multocida*	59897.6 ± 12.81	1247 ± 4.65
(3) LPS and *P. multocida *simultaneously	1024041.1 ± 32.41	2080027 ± 89.50
(4) LPS and 30 min later *P. multocida *	1020481.7 ± 1188.70	2079876 ± 333.80
(5)* P. multocida *and 30 min later its LPS	634092.6 ± 6.94	31586 ± 4.80
(6) LPS *P. multocida*	0	1998847 ± 550.56
